# Supplementary material for: Effects of jockey position and surfaces on horse movement asymmetry and horse-jockey synchronisation during trotting exercise
Source: PLoS One. 2025 Jun 2;20(5):e0324753. doi: 10.1371/journal.pone.0324753 (PMC12129221; doi:10.1371/journal.pone.0324753)
Supplement: S1 File — (DOCX) [file pone.0324753.s001.docx]

# Supplementary Material

## S.1 Data Tables

### S.1.1 Horse Stance and Flight timings

**Table S1** Significance values from the Linear Mixed Models for time-offsets between the two mid-stance phases and the two mid-flight phases in the trot stride cycle.

| **Time point** | **Fixed factor** | **F** |  | **P value** |
| --- | --- | --- | --- | --- |
| Mid-stance | Surface | 1.01 |  | 0.371 |
|  | Jockey position | 21.63 |  | <0.001 |
|  | Stride time | 0.05 |  | 0.830 |
| Mid-flight | Surface | 0.32 |  | 0.725 |
|  | Jockey position | 6.38 |  | 0.015 |
|  | Stride time | 0.43 |  | 0.529 |

**Table S2** Estimated Marginal Means for surface effects on time-offsets between the two mid-stance phases and the two mid-flight phases in the trot stride cycle.

| **Time point** | **Surface** | **Mean** | **Std. Error** | **df** | **95% Confidence Interval (lower bound)** | **95% Confidence Interval (upper bound)** |
| --- | --- | --- | --- | --- | --- | --- |
| Mid-stance | Artificial | 48.8 | 0.3 | 21.0 | 48.1 | 49.4 |
|  | Grass | 48.9 | 0.3 | 20.7 | 48.2 | 49.6 |
|  | Tarmac | 48.3 | 0.4 | 19.2 | 47.5 | 49.0 |
| Mid-flight | Artificial | 50.2 | 0.3 | 28.6 | 49.6 | 50.8 |
|  | Grass | 50.0 | 0.3 | 28.1 | 49.4 | 50.6 |
|  | Tarmac | 50.4 | 0.3 | 24.8 | 49.7 | 51.0 |

**Table S3** Estimated Marginal Means for jockey position effects on time-offsets between the two mid-stance phases and the two mid-flight phases in the trot stride cycle.

| **Time point** | **Jockey position** | **Mean** | **Std. Error** | **df** | **95% Confidence Interval (lower bound)** | **95% Confidence Interval (upper bound)** |
| --- | --- | --- | --- | --- | --- | --- |
| Mid-stance | Rising | 47.8 | 0.2 | 7.5 | 47.2 | 48.3 |
|  | Two-point | 49.5 | 0.3 | 19.6 | 48.8 | 50.2 |
| Mid-flight | Rising | 49.7 | 0.2 | 9.5 | 49.3 | 50.2 |
|  | Two-point | 50.6 | 0.3 | 26.6 | 50.0 | 51.2 |

**Table S4** Estimated Marginal Means for surface and jockey position effects on time-offsets between the two mid-stance phases and the two mid-flight phases in the trot stride cycle.

| **Time point** | **Surface** | **Jockey position** | **Mean** | **Std. Error** | **df** | **95% Confidence Interval (lower bound)** | **95% Confidence Interval (upper bound)** |
| --- | --- | --- | --- | --- | --- | --- | --- |
| Mid-stance | Artificial | Rising | 47.9 | 0.4 | 23.3 | 47.1 | 48.6 |
|  |  | Two-point | 49.6 | 0.4 | 34.5 | 48.8 | 50.5 |
|  | Grass | Rising | 48.0 | 0.4 | 22.8 | 47.3 | 48.8 |
|  |  | Two-point | 49.8 | 0.4 | 34.5 | 49.0 | 50.7 |
|  | Tarmac | Rising | 47.4 | 0.4 | 23.1 | 46.6 | 48.1 |
|  |  | Two-point | 49.2 | 0.4 | 28.9 | 48.2 | 50.1 |
| Mid-flight | Artificial | Rising | 49.7 | 0.3 | 30.7 | 49.1 | 50.4 |
|  |  | Two-point | 50.6 | 0.4 | 41.9 | 49.9 | 51.4 |
|  | Grass | Rising | 49.6 | 0.3 | 29.7 | 48.9 | 50.2 |
|  |  | Two-point | 50.5 | 0.4 | 41.9 | 49.7 | 51.2 |
|  | Tarmac | Rising | 49.9 | 0.3 | 30.4 | 49.3 | 50.6 |
|  |  | Two-point | 50.8 | 0.4 | 34.7 | 50.0 | 51.6 |

### S1.1.2 Horse Symmetry

**Table S5** Significance values from the Linear Mixed Models for horse movement asymmetry at the poll, withers and sacrum.

| **Anatomical location** | **Displacement parameter** | **Fixed factor** | **F value** | **P value** |
| --- | --- | --- | --- | --- |
| Poll | MinDiff | Surface | 2.45 | 0.099 |
|  |  | Jockey position | 6.17 | 0.005 |
|  |  | Stride time | 0.05 | 0.830 |
|  |  | Surface * Stride time | 2.36 | 0.107 |
|  |  | Jockey position * Stride time | 5.54 | 0.008 |
|  | MaxDiff | Surface | 0.75 | 0.477 |
|  |  | Jockey position | 1.74 | 0.187 |
|  |  | Stride time | 0.00 | 0.949 |
|  | UpDiff | Surface | 1.73 | 0.189 |
|  |  | Jockey position | 0.44 | 0.649 |
|  |  | Stride time | 0.09 | 0.770 |
| Withers | MinDiff | Surface | 3.46 | 0.042 |
|  |  | Jockey position | 4.25 | 0.021 |
|  |  | Stride time | 0.00 | 0.965 |
|  |  | Surface * Stride time | 3.26 | 0.049 |
|  |  | Jockey position * Stride time | 4.18 | 0.023 |
|  | MaxDiff | Surface | 1.80 | 0.178 |
|  |  | Jockey position | 8.00 | 0.001 |
|  |  | Stride time | 0.79 | 0.379 |
|  |  | Jockey position * Stride time | 5.49 | 0.008 |
|  | UpDiff | Surface | 0.58 | 0.562 |
|  |  | Jockey position | 7.91 | 0.001 |
|  |  | Stride time | 1.00 | 0.323 |
|  |  | Jockey position * Stride time | 6.28 | 0.004 |
| Sacrum | MinDiff | Surface | 0.87 | 0.427 |
|  |  | Jockey position | 1.38 | 0.263 |
|  |  | Stride time | 1.48 | 0.230 |
|  | MaxDiff | Surface | 3.75 | 0.031 |
|  |  | Jockey position | 67.33 | <0.001 |
|  |  | Stride time | 0.96 | 0.333 |
|  | UpDiff | Surface | 2.64 | 0.083 |
|  |  | Jockey position | 67.85 | <0.001 |
|  |  | Stride time | 7.22 | 0.010 |

**Table S6** Estimated Marginal Means for surface effects on horse movement asymmetry at the poll, withers and sacrum.

| **Anatomical location** | **Displacement parameter** | **Surface** | **Mean** | **Std. Error** | **df** | **95% Confidence Interval (lower bound)** | **95% Confidence Interval (upper bound)** |
| --- | --- | --- | --- | --- | --- | --- | --- |
| Poll | MinDiff | Artificial | -7.2 | 3.6 | 8.1 | -15.4 | 1.1 |
|  |  | Grass | -10.9 | 3.6 | 8.3 | -19.2 | -2.7 |
|  |  | Tarmac | -9.2 | 4.0 | 11.9 | -18.0 | -0.4 |
|  | MaxDiff | Artificial | -15.1 | 8.9 | 6.2 | -36.7 | 6.6 |
|  |  | Grass | -9.8 | 9.0 | 6.3 | -31.5 | 11.9 |
|  |  | Tarmac | -9.5 | 9.1 | 6.7 | -31.3 | 12.3 |
|  | UpDiff | Artificial | 13.2 | 9.6 | 6.1 | -10.3 | 36.6 |
|  |  | Grass | 4.8 | 9.7 | 6.2 | -18.7 | 28.2 |
|  |  | Tarmac | 4.4 | 9.8 | 6.5 | -19.2 | 27.9 |
| Withers | MinDiff | Artificial | -1.8 | 2.1 | 5.0 | -7.2 | 3.6 |
|  |  | Grass | -3.4 | 2.1 | 5.0 | -8.8 | 2.0 |
|  |  | Tarmac | -1.8 | 2.2 | 6.0 | -7.3 | 3.6 |
|  | MaxDiff | Artificial | 2.6 | 3.6 | 5.3 | -6.4 | 11.6 |
|  |  | Grass | 0.8 | 3.6 | 5.3 | -8.3 | 9.8 |
|  |  | Tarmac | 0.0 | 3.6 | 5.5 | -9.1 | 9.0 |
|  | UpDiff | Artificial | -2.7 | 6.0 | 5.0 | -18.1 | 12.8 |
|  |  | Grass | -4.7 | 6.0 | 5.0 | -20.2 | 10.8 |
|  |  | Tarmac | -2.8 | 6.1 | 5.1 | -18.2 | 12.7 |
| Sacrum | MinDiff | Artificial | 2.5 | 2.2 | 5.4 | -3.1 | 8.1 |
|  |  | Grass | 1.3 | 2.2 | 5.5 | -4.3 | 6.9 |
|  |  | Tarmac | 1.1 | 2.3 | 5.8 | -4.5 | 6.7 |
|  | MaxDiff | Artificial | -0.4 | 2.2 | 6.6 | -5.7 | 4.9 |
|  |  | Grass | -0.6 | 2.2 | 6.8 | -5.9 | 4.7 |
|  |  | Tarmac | 4.5 | 2.3 | 7.3 | -0.9 | 9.8 |
|  | UpDiff | Artificial | -2.9 | 3.7 | 5.8 | -12.0 | 6.2 |
|  |  | Grass | -4.8 | 3.7 | 5.9 | -13.8 | 4.3 |
|  |  | Tarmac | 0.5 | 3.7 | 6.2 | -8.6 | 9.6 |

**Table S7** Estimated Marginal Means for jockey position effects on horse movement asymmetry at the poll, withers and sacrum.

| **Anatomical Location** | **Displacement parameter** | **Jockey position** | **Mean** | **Std. Error** | **df** | **95% Confidence Interval (lower bound)** | **95% Confidence Interval (upper bound)** |
| --- | --- | --- | --- | --- | --- | --- | --- |
| Poll | MinDiff | Rising LD | -1.8 | 3.6 | 8.3 | -10.0 | 6.4 |
|  |  | Rising RD | -16.0 | 3.6 | 8.2 | -24.2 | -7.8 |
|  |  | Two-point | -9.5 | 3.7 | 9.0 | -17.8 | -1.2 |
|  | MaxDiff | Rising LD | -14.3 | 8.9 | 6.2 | -36.0 | 7.4 |
|  |  | Rising RD | -6.0 | 8.9 | 6.2 | -27.7 | 15.6 |
|  |  | Two-point | -14.0 | 9.0 | 6.3 | -35.7 | 7.7 |
|  | UpDiff | Rising LD | 9.2 | 9.6 | 6.1 | -14.2 | 32.7 |
|  |  | Rising RD | 4.7 | 9.6 | 6.0 | -18.8 | 28.1 |
|  |  | Two-point | 8.4 | 9.6 | 6.2 | -15.0 | 31.9 |
| Withers | MinDiff | Rising LD | -3.1 | 2.1 | 5.1 | -8.5 | 2.3 |
|  |  | Rising RD | -2.5 | 2.1 | 4.9 | -7.9 | 3.0 |
|  |  | Two-point | -1.5 | 2.1 | 5.2 | -6.9 | 3.9 |
|  | MaxDiff | Rising LD | -9.8 | 3.6 | 5.3 | -18.8 | -0.8 |
|  |  | Rising RD | 12.5 | 3.6 | 5.3 | 3.5 | 21.5 |
|  |  | Two-point | 0.6 | 3.6 | 5.4 | -8.4 | 9.6 |
|  | UpDiff | Rising LD | -14.6 | 6.0 | 5.0 | -30.0 | 0.9 |
|  |  | Rising RD | 7.9 | 6.0 | 4.9 | -7.6 | 23.3 |
|  |  | Two-point | -3.4 | 6.0 | 5.0 | -18.9 | 12.0 |
| Sacrum | MinDiff | Rising LD | 2.5 | 2.2 | 5.4 | -3.1 | 8.2 |
|  |  | Rising RD | 0.7 | 2.2 | 5.5 | -4.9 | 6.3 |
|  |  | Two-point | 1.7 | 2.2 | 5.5 | -3.9 | 7.3 |
|  | MaxDiff | Rising LD | 11.0 | 2.2 | 6.5 | 5.7 | 16.2 |
|  |  | Rising RD | -9.4 | 2.2 | 6.6 | -14.7 | -4.1 |
|  |  | Two-point | 2.0 | 2.2 | 6.7 | -3.3 | 7.2 |
|  | UpDiff | Rising LD | 7.8 | 3.7 | 5.8 | -1.3 | 16.9 |
|  |  | Rising RD | -14.7 | 3.7 | 5.8 | -23.7 | -5.6 |
|  |  | Two-point | -0.3 | 3.7 | 5.9 | -9.3 | 8.8 |

**Table S8** Estimated Marginal Means for surface and jockey position effects on horse movement asymmetry at the poll, withers and sacrum.

| **Anatomical Location** | **Displacement parameter** | **Surface** | **Jockey position** | **Mean** | **Std. Error** | **df** | **95% Confidence Interval**  **(lower bound)** | **95% Confidence Interval**  **(upper bound)** |
| --- | --- | --- | --- | --- | --- | --- | --- | --- |
| Poll | MinDiff | Artificial | Rising LD | 0.1 | 4.0 | 12.0 | -8.6 | 8.8 |
|  |  |  | Rising RD | -14.1 | 4.0 | 12.1 | -22.8 | -5.4 |
|  |  |  | Two-point | -7.6 | 4.0 | 11.9 | -16.2 | 1.1 |
|  |  | Grass | Rising LD | -3.6 | 4.0 | 12.3 | -12.4 | 5.1 |
|  |  |  | Rising RD | -17.9 | 4.0 | 12.3 | -26.6 | -9.1 |
|  |  |  | Two-point | -11.3 | 4.0 | 12.1 | -20.0 | -2.7 |
|  |  | Tarmac | Rising LD | -1.9 | 4.3 | 15.4 | -11.1 | 7.3 |
|  |  |  | Rising RD | -16.1 | 4.3 | 14.9 | -25.2 | -7.0 |
|  |  |  | Two-point | -9.6 | 4.6 | 17.4 | -19.4 | 0.2 |
|  | MaxDiff | Artificial | Rising LD | -17.9 | 9.4 | 7.7 | -39.9 | 4.0 |
|  |  |  | Rising RD | -9.7 | 9.4 | 7.7 | -31.6 | 12.3 |
|  |  |  | Two-point | -17.6 | 9.4 | 7.4 | -39.5 | 4.3 |
|  |  | Grass | Rising LD | -12.6 | 9.5 | 7.8 | -34.6 | 9.4 |
|  |  |  | Rising RD | -4.4 | 9.5 | 7.9 | -26.4 | 17.6 |
|  |  |  | Two-point | -12.3 | 9.4 | 7.4 | -34.2 | 9.5 |
|  |  | Tarmac | Rising LD | -12.3 | 9.5 | 7.7 | -34.3 | 9.6 |
|  |  |  | Rising RD | -4.1 | 9.5 | 7.7 | -26.0 | 17.9 |
|  |  |  | Two-point | -12.0 | 9.8 | 8.8 | -34.4 | 10.3 |
|  | UpDiff | Artificial | Rising LD | 15.0 | 10.1 | 7.5 | -8.7 | 38.7 |
|  |  |  | Rising RD | 10.4 | 10.1 | 7.4 | -13.2 | 34.1 |
|  |  |  | Two-point | 14.2 | 10.0 | 7.2 | -9.4 | 37.7 |
|  |  | Grass | Rising LD | 6.5 | 10.2 | 7.6 | -17.2 | 30.3 |
|  |  |  | Rising RD | 2.0 | 10.2 | 7.6 | -21.7 | 25.7 |
|  |  |  | Two-point | 5.7 | 10.0 | 7.2 | -17.9 | 29.3 |
|  |  | Tarmac | Rising LD | 6.2 | 10.1 | 7.5 | -17.5 | 29.9 |
|  |  |  | Rising RD | 1.6 | 10.2 | 7.5 | -22.1 | 25.3 |
|  |  |  | Two-point | 5.3 | 10.5 | 8.5 | -18.7 | 29.4 |
| Withers | MinDiff | Artificial | Rising LD | -2.5 | 2.2 | 5.9 | -7.9 | 2.9 |
|  |  |  | Rising RD | -1.9 | 2.2 | 5.8 | -7.3 | 3.5 |
|  |  |  | Two-point | -1.0 | 2.2 | 5.7 | -6.4 | 4.4 |
|  |  | Grass | Rising LD | -4.1 | 2.2 | 6.1 | -9.5 | 1.3 |
|  |  |  | Rising RD | -3.5 | 2.2 | 5.8 | -8.9 | 1.9 |
|  |  |  | Two-point | -2.6 | 2.2 | 5.8 | -8.0 | 2.8 |
|  |  | Tarmac | Rising LD | -2.6 | 2.3 | 6.7 | -8.0 | 2.8 |
|  |  |  | Rising RD | -2.0 | 2.3 | 6.5 | -7.4 | 3.5 |
|  |  |  | Two-point | -1.0 | 2.4 | 7.6 | -6.5 | 4.5 |
|  | MaxDiff | Artificial | Rising LD | -8.3 | 3.7 | 5.8 | -17.3 | 0.7 |
|  |  |  | Rising RD | 14.0 | 3.7 | 5.8 | 5.0 | 23.0 |
|  |  |  | Two-point | 2.1 | 3.6 | 5.8 | -6.9 | 11.1 |
|  |  | Grass | Rising LD | -10.1 | 3.7 | 5.9 | -19.2 | -1.1 |
|  |  |  | Rising RD | 12.1 | 3.7 | 5.9 | 3.1 | 21.1 |
|  |  |  | Two-point | 0.3 | 3.6 | 5.8 | -8.7 | 9.3 |
|  |  | Tarmac | Rising LD | -10.9 | 3.7 | 5.9 | -19.9 | -1.9 |
|  |  |  | Rising RD | 11.3 | 3.7 | 5.9 | 2.3 | 20.4 |
|  |  |  | Two-point | -0.5 | 3.7 | 6.4 | -9.6 | 8.5 |
|  | UpDiff | Artificial | Rising LD | -13.8 | 6.1 | 5.4 | -29.3 | 1.6 |
|  |  |  | Rising RD | 8.6 | 6.1 | 5.4 | -6.9 | 24.0 |
|  |  |  | Two-point | -2.7 | 6.1 | 5.4 | -18.2 | 12.7 |
|  |  | Grass | Rising LD | -15.9 | 6.2 | 5.5 | -31.3 | -0.5 |
|  |  |  | Rising RD | 6.5 | 6.1 | 5.4 | -8.9 | 21.9 |
|  |  |  | Two-point | -4.8 | 6.1 | 5.3 | -20.2 | 10.7 |
|  |  | Tarmac | Rising LD | -13.9 | 6.1 | 5.4 | -29.4 | 1.5 |
|  |  |  | Rising RD | 8.5 | 6.2 | 5.5 | -6.9 | 23.9 |
|  |  |  | Two-point | -2.8 | 6.3 | 5.9 | -18.2 | 12.6 |
| Sacrum | MinDiff | Artificial | Rising LD | 3.4 | 2.3 | 6.3 | -2.2 | 9.0 |
|  |  |  | Rising RD | 1.6 | 2.4 | 6.6 | -4.1 | 7.2 |
|  |  |  | Two-point | 2.5 | 2.3 | 6.3 | -3.1 | 8.2 |
|  |  | Grass | Rising LD | 2.2 | 2.3 | 6.4 | -3.4 | 7.9 |
|  |  |  | Rising RD | 0.4 | 2.4 | 6.8 | -5.3 | 6.0 |
|  |  |  | Two-point | 1.4 | 2.3 | 6.3 | -4.3 | 7.0 |
|  |  | Tarmac | Rising LD | 2.0 | 2.4 | 6.7 | -3.6 | 7.6 |
|  |  |  | Rising RD | 0.1 | 2.3 | 6.4 | -5.5 | 5.8 |
|  |  |  | Two-point | 1.1 | 2.4 | 7.3 | -4.6 | 6.8 |
|  | MaxDiff | Artificial | Rising LD | 9.4 | 2.4 | 9.7 | 3.9 | 14.9 |
|  |  |  | Rising RD | -11.0 | 2.5 | 9.9 | -16.5 | -5.5 |
|  |  |  | Two-point | 0.4 | 2.4 | 9.5 | -5.0 | 5.8 |
|  |  | Grass | Rising LD | 9.2 | 2.5 | 10.0 | 3.7 | 14.7 |
|  |  |  | Rising RD | -11.2 | 2.5 | 10.2 | -16.7 | -5.6 |
|  |  |  | Two-point | 0.2 | 2.4 | 9.5 | -5.2 | 5.6 |
|  |  | Tarmac | Rising LD | 14.3 | 2.5 | 10.0 | 8.8 | 19.8 |
|  |  |  | Rising RD | -6.1 | 2.4 | 9.8 | -11.6 | -0.6 |
|  |  |  | Two-point | 5.3 | 2.6 | 11.4 | -0.5 | 11.0 |
|  | UpDiff | Artificial | Rising LD | 7.3 | 3.9 | 7.0 | -1.8 | 16.4 |
|  |  |  | Rising RD | -15.2 | 3.9 | 7.2 | -24.3 | -6.1 |
|  |  |  | Two-point | -0.8 | 3.8 | 6.9 | -9.9 | 8.3 |
|  |  | Grass | Rising LD | 5.4 | 3.9 | 7.1 | -3.7 | 14.5 |
|  |  |  | Rising RD | -17.1 | 3.9 | 7.4 | -26.2 | -7.9 |
|  |  |  | Two-point | -2.6 | 3.8 | 6.9 | -11.7 | 6.5 |
|  |  | Tarmac | Rising LD | 10.7 | 3.9 | 7.2 | 1.6 | 19.8 |
|  |  |  | Rising RD | -11.8 | 3.9 | 7.0 | -20.9 | -2.6 |
|  |  |  | Two-point | 2.7 | 4.0 | 8.0 | -6.6 | 11.9 |

### S1.1.2 Time lags between vertical horse and jockey upper body displacements

**Table S9** Significance values from the Linear Mixed Models for time lags between horse and jockey vertical displacements assessed around the stance and flight phases.

| **Jockey anatomical site** | **Stride stage** | **Fixed factor** | **F value** | **P value** |
| --- | --- | --- | --- | --- |
| Pelvis | Stance | Surface | 4.78 | 0.011 |
|  |  | Jockey position | 9.23 | <0.001 |
|  |  | Stride time | 44.24 | <0.001 |
|  |  | Jockey position * Stride time | 5.63 | 0.005 |
|  | Flight | Surface | 3.58 | 0.032 |
|  |  | Jockey position | 22.40 | <0.001 |
|  |  | Stride time | 4.65 | 0.038 |
|  |  | Jockey position * Stride time | 13.13 | <0.001 |
| Mid-back | Stance | Surface | 3.88 | 0.024 |
|  |  | Jockey position | 211.94 | <0.001 |
|  |  | Stride time | 35.62 | <0.001 |
|  | Flight | Surface | 1.73 | 0.182 |
|  |  | Jockey position | 29.83 | <0.001 |
|  |  | Stride time | 3.44 | 0.073 |
|  |  | Surface * Jockey position | 2.42 | 0.054 |
|  |  | Jockey position * Stride time | 15.15 | <0.001 |
| Upper-back | Stance | Surface | 5.50 | 0.005 |
|  |  | Jockey position | 12.07 | <0.001 |
|  |  | Stride time | 21.23 | <0.001 |
|  |  | Jockey position * Stride time | 8.61 | <0.001 |
|  | Flight | Surface | 2.81 | 0.065 |
|  |  | Jockey position | 39.98 | <0.001 |
|  |  | Stride time | 2.82 | 0.104 |
|  |  | Jockey position * Stride time | 21.62 | <0.001 |

**Table S10** Estimated Marginal Means for surface effects on time lags between horse and jockey vertical displacements around the stance and flight phases.

| **Jockey anatomical site** | **Stride stage** | **Surface** | **Mean** | **Std. Error** | **df** | **95% Confidence Interval (lower bound)** | **95% Confidence Interval (upper bound)** |
| --- | --- | --- | --- | --- | --- | --- | --- |
| Pelvis | Stance | Artificial | -3.4 | 0.2 | 18.9 | -3.7 | -3.0 |
|  |  | Grass | -3.1 | 0.2 | 18.8 | -3.5 | -2.8 |
|  |  | Tarmac | -2.7 | 0.2 | 18.1 | -3.1 | -2.3 |
|  | Flight | Artificial | -3.7 | 0.4 | 9.1 | -4.6 | -2.7 |
|  |  | Grass | -3.7 | 0.4 | 9.3 | -4.6 | -2.7 |
|  |  | Tarmac | -2.6 | 0.4 | 10.0 | -3.6 | -1.6 |
| Mid-back | Stance | Artificial | -3.4 | 0.2 | 19.0 | -3.7 | -3.0 |
|  |  | Grass | -3.1 | 0.2 | 18.7 | -3.4 | -2.7 |
|  |  | Tarmac | -2.7 | 0.2 | 18.0 | -3.1 | -2.4 |
|  | Flight | Artificial | -4.0 | 0.3 | 9.4 | -4.7 | -3.4 |
|  |  | Grass | -3.9 | 0.3 | 9.6 | -4.5 | -3.2 |
|  |  | Tarmac | -3.4 | 0.3 | 10.2 | -4.1 | -2.7 |
| Upper-back | Stance | Artificial | -3.7 | 0.2 | 10.8 | -4.2 | -3.3 |
|  |  | Grass | -3.4 | 0.2 | 11.0 | -3.9 | -3.0 |
|  |  | Tarmac | -3.0 | 0.2 | 11.8 | -3.5 | -2.5 |
|  | Flight | Artificial | -3.9 | 0.3 | 10.3 | -4.7 | -3.1 |
|  |  | Grass | -3.7 | 0.3 | 10.5 | -4.5 | -2.9 |
|  |  | Tarmac | -3.0 | 0.4 | 11.1 | -3.8 | -2.2 |

**Table S11** Estimated Marginal Means for jockey position effects on time lags between horse and jockey vertical displacements around the stance and flight phases.

| **Jockey anatomical site** | **Stride stage** | **Jockey position** | **Mean** | **Std. Error** | **df** | **95% Confidence Interval**  **(lower bound)** | **95% Confidence Interval**  **(upper bound)** |
| --- | --- | --- | --- | --- | --- | --- | --- |
| Pelvis | Stance | Rising trot- StanceTDiff seated | -0.4 | 0.2 | 18.9 | -0.7 | -0.1 |
|  |  | Rising trot - StanceTDiff standing | -5.0 | 0.2 | 18.9 | -5.3 | -4.6 |
|  |  | Two-point - StanceTDiff | -3.8 | 0.2 | 19.8 | -4.2 | -3.5 |
|  | Flight | Rising trot - FlightTDiff post-seated | -9.3 | 0.4 | 9.0 | -10.3 | -8.3 |
|  |  | Rising trot - FlightTDiff post-standing | 4.9 | 0.4 | 9.0 | 3.9 | 5.8 |
|  |  | Two-point - FlightTDiff | -5.5 | 0.4 | 9.6 | -6.5 | -4.5 |
| Mid-back | Stance | Rising trot - StanceTDiff seated | -0.6 | 0.2 | 19.1 | -0.9 | -0.2 |
|  |  | Rising trot - StanceTDiff standing | -4.5 | 0.2 | 19.1 | -4.8 | -4.2 |
|  |  | Two-point - StanceTDiff | -4.1 | 0.2 | 18.8 | -4.4 | -3.7 |
|  | Flight | Rising trot - FlightTDiff post-seated | -10.4 | 0.3 | 9.3 | -11.0 | -9.7 |
|  |  | Rising trot - FlightTDiff post-standing | 5.3 | 0.3 | 9.3 | 4.7 | 6.0 |
|  |  | Two-point - FlightTDiff | -6.3 | 0.3 | 10.0 | -7.0 | -5.6 |
| Upper-back | Stance | Rising trot - StanceTDiff seated | -1.1 | 0.2 | 10.7 | -1.5 | -0.6 |
|  |  | Rising trot - StanceTDiff standing | -4.0 | 0.2 | 10.7 | -4.4 | -3.5 |
|  |  | Two-point - StanceTDiff | -5.1 | 0.2 | 11.5 | -5.6 | -4.6 |
|  | Flight | Rising trot - FlightTDiff post-seated | -11.0 | 0.3 | 10.2 | -11.7 | -10.2 |
|  |  | Rising trot - FlightTDiff post-standing | 7.3 | 0.3 | 10.2 | 6.6 | 8.1 |
|  |  | Two-point - FlightTDiff | -7.0 | 0.4 | 10.9 | -7.7 | -6.2 |

**Table S12** Estimated Marginal Means for surface and jockey position effects on time lags between horse and jockey vertical displacements around the stance and flight phases.

| **Jockey anatomical site** | **Stride stage** | **Surface** | **Jockey position** | **Mean** | **Std. Error** | **df** | **95% Confidence Interval (lower bound)** | **95% Confidence Interval (upper bound)** |
| --- | --- | --- | --- | --- | --- | --- | --- | --- |
| Pelvis | Stance | Artificial | Rising trot - StanceTDiff seated | -0.7 | 0.2 | 36.2 | -1.1 | -0.3 |
|  |  |  | Rising trot - StanceTDiff standing | -5.3 | 0.2 | 36.2 | -5.7 | -4.9 |
|  |  |  | Two-point - StanceTDiff | -4.1 | 0.2 | 38.9 | -4.5 | -3.7 |
|  |  | Grass | Rising trot - StanceTDiff seated | -0.5 | 0.2 | 35.3 | -0.9 | 0.0 |
|  |  |  | Rising trot - StanceTDiff standing | -5.0 | 0.2 | 35.3 | -5.4 | -4.6 |
|  |  |  | Two-point - StanceTDiff | -3.9 | 0.2 | 39.2 | -4.3 | -3.5 |
|  |  | Tarmac | Rising trot- StanceTDiff seated | 0.0 | 0.2 | 35.7 | -0.5 | 0.4 |
|  |  |  | Rising trot- StanceTDiff standing | -4.6 | 0.2 | 35.7 | -5.0 | -4.2 |
|  |  |  | Two-point - StanceTDiff | -3.5 | 0.2 | 31.4 | -3.9 | -3.0 |
|  | Flight | Artificial | Rising trot - FlightTDiff post-seated | -9.6 | 0.5 | 14.8 | -10.7 | -8.6 |
|  |  |  | Rising trot - FlightTDiff post-standing | 4.5 | 0.5 | 14.8 | 3.5 | 5.6 |
|  |  |  | Two-point - FlightTDiff | -5.9 | 0.5 | 14.9 | -6.9 | -4.8 |
|  |  | Grass | Rising trot - FlightTDiff post-seated | -9.7 | 0.5 | 15.1 | -10.7 | -8.6 |
|  |  |  | Rising trot - FlightTDiff post-standing | 4.5 | 0.5 | 15.1 | 3.5 | 5.6 |
|  |  |  | Two-point - FlightTDiff | -5.9 | 0.5 | 14.9 | -6.9 | -4.9 |
|  |  | Tarmac | Rising trot - FlightTDiff post-seated | -8.6 | 0.5 | 14.9 | -9.6 | -7.5 |
|  |  |  | Rising trot - FlightTDiff post-standing | 5.6 | 0.5 | 14.9 | 4.5 | 6.6 |
|  |  |  | Two-point - FlightTDiff | -4.8 | 0.5 | 16.9 | -5.9 | -3.7 |
| Mid-back | Stance | Artificial | Rising trot- StanceTDiff seated | -0.9 | 0.2 | 37.4 | -1.3 | -0.5 |
|  |  |  | Rising trot- StanceTDiff standing | -4.8 | 0.2 | 37.4 | -5.2 | -4.4 |
|  |  |  | Two-point - StanceTDiff | -4.4 | 0.2 | 39.4 | -4.8 | -4.0 |
|  |  | Grass | Rising trot- StanceTDiff seated | -0.6 | 0.2 | 36.3 | -1.0 | -0.2 |
|  |  |  | Rising trot- StanceTDiff standing | -4.5 | 0.2 | 36.3 | -4.9 | -4.1 |
|  |  |  | Two-point - StanceTDiff | -4.1 | 0.2 | 39.4 | -4.5 | -3.7 |
|  |  | Tarmac | Rising trot- StanceTDiff seated | -0.2 | 0.2 | 37.0 | -0.7 | 0.2 |
|  |  |  | Rising trot- StanceTDiff standing | -4.2 | 0.2 | 37.0 | -4.6 | -3.8 |
|  |  |  | Two-point - StanceTDiff | -3.8 | 0.2 | 31.3 | -4.2 | -3.3 |
|  | Flight | Artificial | Rising trot - FlightTDiff post-seated | -10.6 | 0.4 | 30.9 | -11.5 | -9.8 |
|  |  |  | Rising trot - FlightTDiff post-standing | 5.2 | 0.4 | 30.9 | 4.4 | 6.1 |
|  |  |  | Two-point - FlightTDiff | -6.7 | 0.4 | 29.9 | -7.5 | -5.9 |
|  |  | Grass | Rising trot - FlightTDiff post-seated | -9.9 | 0.4 | 31.7 | -10.8 | -9.0 |
|  |  |  | Rising trot - FlightTDiff post-standing | 4.6 | 0.4 | 31.7 | 3.7 | 5.5 |
|  |  |  | Two-point - FlightTDiff | -6.3 | 0.4 | 29.8 | -7.2 | -5.5 |
|  |  | Tarmac | Rising trot - FlightTDiff post-seated | -10.6 | 0.4 | 31.3 | -11.4 | -9.7 |
|  |  |  | Rising trot - FlightTDiff post-standing | 6.2 | 0.4 | 31.3 | 5.3 | 7.0 |
|  |  |  | Two-point - FlightTDiff | -5.9 | 0.5 | 35.2 | -6.9 | -5.0 |
| Upper-back | Stance | Artificial | Rising trot- StanceTDiff seated | -1.4 | 0.2 | 18.4 | -1.9 | -0.9 |
|  |  |  | Rising trot- StanceTDiff standing | -4.3 | 0.2 | 18.4 | -4.8 | -3.8 |
|  |  |  | Two-point - StanceTDiff | -5.5 | 0.2 | 18.6 | -6.0 | -5.0 |
|  |  | Grass | Rising trot- StanceTDiff seated | -1.1 | 0.2 | 18.6 | -1.6 | -0.6 |
|  |  |  | Rising trot- StanceTDiff standing | -4.0 | 0.2 | 18.6 | -4.5 | -3.5 |
|  |  |  | Two-point - StanceTDiff | -5.2 | 0.2 | 18.7 | -5.7 | -4.6 |
|  |  | Tarmac | Rising trot- StanceTDiff seated | -0.7 | 0.2 | 18.5 | -1.2 | -0.2 |
|  |  |  | Rising trot- StanceTDiff standing | -3.6 | 0.2 | 18.5 | -4.1 | -3.0 |
|  |  |  | Two-point - StanceTDiff | -4.7 | 0.3 | 20.2 | -5.3 | -4.2 |
|  | Flight | Artificial | Rising trot - FlightTDiff post-seated | -11.3 | 0.4 | 17.7 | -12.2 | -10.5 |
|  |  |  | Rising trot - FlightTDiff post-standing | 7.0 | 0.4 | 17.7 | 6.1 | 7.8 |
|  |  |  | Two-point - FlightTDiff | -7.3 | 0.4 | 18.0 | -8.2 | -6.5 |
|  |  | Grass | Rising trot - FlightTDiff post-seated | -11.1 | 0.4 | 17.9 | -12.0 | -10.3 |
|  |  |  | Rising trot - FlightTDiff post-standing | 7.2 | 0.4 | 17.9 | 6.3 | 8.0 |
|  |  |  | Two-point - FlightTDiff | -7.1 | 0.4 | 18.1 | -8.0 | -6.3 |
|  |  | Tarmac | Rising trot - FlightTDiff post-seated | -10.5 | 0.4 | 17.8 | -11.3 | -9.6 |
|  |  |  | Rising trot - FlightTDiff post-standing | 7.9 | 0.4 | 17.8 | 7.0 | 8.7 |
|  |  |  | Two-point - FlightTDiff | -6.4 | 0.4 | 19.4 | -7.4 | -5.5 |

## S.2 Stride time variability

Figure S1 illustrates the distribution in stride times across conditions.


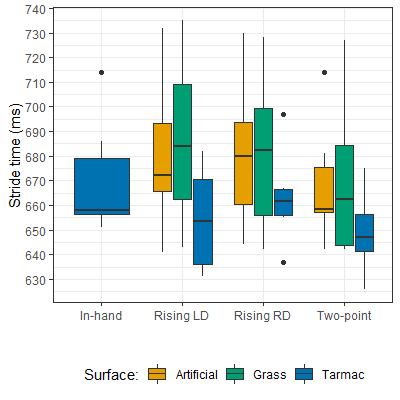


**Fig S1****. Distribution of stride times across trial conditions.** LD = left diagonal. RD = right diagonal.

## S.3 Jockey trunk roll angles

Three linear mixed models were run in SPPS to compare jockey trunk roll angles at the pelvis, mid-back and upper-back in rising trot and two-point seat across the three surface types. Average roll angles were computed across the full stride cycle in each case per condition. Surface (tarmac, artificial or grass) and jockey position (rising or two-point seat) were included as fixed factors. Horse was included as a random factor. Stride time was included as a covariate. Interaction terms between surface, jockey position and stride time were included also. Data from the two types of rising trial (left and right diagonal) were combined and stride times for the two conditions averaged in each instance. The significance threshold in all statistical tests was set at p<0.05. Data are summarised in Figure S2.


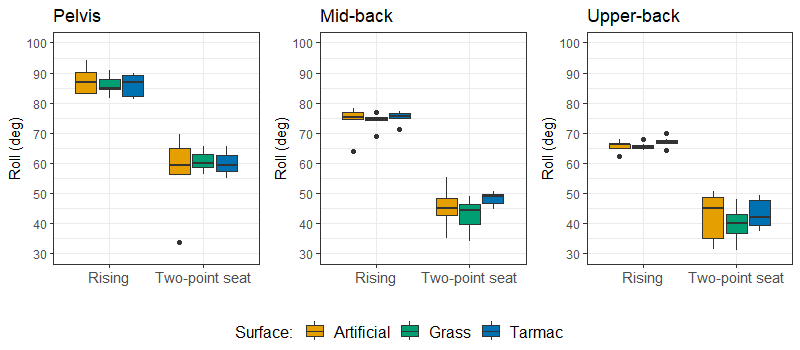
**Fig S2. Jockey roll angles at their pelvis, mid-back and upper-back, sub-divided according to riding position (rising trot and two-point seat) and coloured by surface type (artificial, grass and tarmac).**

The roll angle of the sensors positioned at the pelvis, mid-back and upper-back were found to be significantly affected by jockey position (all p<0.001), with roll angles being 1.4–1.6 times larger for the rising position. Surface had no effect on rider roll angles (all p≥0.059) and neither did stride time (p≥0.152), although there was a significant interaction between jockey position and stride time at the upper-back. The broad scale differences between rising and two-point seat positions reflects the more upright posture of the jockey in rising trot. There was also a trend toward decreasing roll angles from pelvis>mid-back>upper-back, indicating the jockey is tipping forwards in their upper body. In the mid and upper-back positions there is a greater spread in the data across all surface types for the two-point seat position, suggesting more instability in jockey position for this condition.
