# Supplementary material for: Effects of jockey position and surfaces on horse movement asymmetry and horse-jockey synchronisation during trotting exercise
Source: PLoS One. 2025 Jun 2;20(5):e0324753. doi: 10.1371/journal.pone.0324753 (PMC12129221; doi:10.1371/journal.pone.0324753)
Supplement: Attachment — Submitted filename: comments.docx [file pone.0324753.s003.docx]

# Title

The current title seems a bit extensive given that your findings indicate surfaces have minimal impact on asymmetry. I recommend rephrasing it to “Effects of the surfaces and jockey position on horse movement asymmetry and horse–jockey synchronization during trotting exercise”.

# Abstract

- **Line 15:** Your opening sentence effectively explains the rationale for the study, but it may give the impression that both horses and jockeys are the primary focus. Consider removing “and jockeys,” and perhaps acknowledge the fact that most injuries occur during canter/gallop exercises. For example “Racehorses can incur injuries not only during gallops, but also during routine trotting exercise to access gallop tracks or warm up.”
- **Line 19:** Here you use the word “turf”, but you call this surface “grass” throughout the whole manuscript. Replace “turf” so it’s not confusing.
- **Line 22–25:** This sentence feels slightly out of place in the abstract. Consider introducing the technical parameters of inertial sensors (in fewer details if you prefer) earlier (around line 17) to improve flow.
- **Line 28–29:** The abbreviations “MinDiff,” “MaxDiff,” and “UpDiff” should be defined if used in the abstract. However, you might consider removing them (and the associated p-values) from this sentence entirely to keep the abstract concise.
- **Line 40:** Adding a single sentence summarizing the results would enhance the abstract’s readability.

# Introduction

- **Line 42:** Specify whose safety you are referring to—jockeys, racehorses, or both.
- **Line 51 and 53:** Using seven or six references to support one statement may be excessive. A common guideline is a maximum of three references per statement. Consider reducing the number of references in this section.
- **Lines 55–72:** This paragraph is somewhat lengthy. Consider shortening by removing some details that are not essential to your analysis.

# Materials and Methods

## Horse and rider participants

- **Line 96:** Include the horses’ ages (e.g., a retired 4-year-old may behave differently than a 15-year-old). Also provide how long they raced and how long they have been retired.
- **Line 96–97:** Since the subject of this sentence is “sample,” the verb should be singular—“was included in this study,” rather than “were included.”
- **Line 97–98:** Consider mentioning the jockey’s experience. How long have they ridden racehorses? Were they a BRS student? Have they ridden in races, or only as a work rider? This information is especially relevant to your discussion in lines 548–550.
- If possible, include details on the saddle type (I imagine it was a racing exercise saddle), including weight. The saddle can significantly influence rising trot technique. Similarly, provide information on stirrup length, since this can affect the rider’s center of gravity.

## Equipment

- This section is the only one that’s numbered. Make sure you maintain consistent formatting.
- Line 101: Maybe add the word “each” to underline that 16 g is the mass of a single sensor, not the collection of all six.
- Line 108: For reproducibility, consider adding basic information about the computer used (operating system, CPU, RAM).

## Trial conditions

- Although optional, including illustrative photos or diagrams of rising trot vs. two-point seat, as well as left- vs. right-diagonal rising trot (similar to your current Figure 1), could help non-specialist readers.
- **Line 120-122:** I understood your point only after reading the whole manuscript, so you may want to rephrase these sentences. Because of the fact the you describe the softness of the artificial and grass (line 120), the following sentence (lines 121-122) sounds a bit like the tarmac was used only as merely a “reference surface” for in-hand trotting.

## Data processing and statistics

### Overview

- **Line 130–131:** Did you consider any interpolation methods other than linear, such as cubic splines or Fourier-based fitting? These might be more appropriate for this kind of wavy function.

### Time between stance and flight phases

- There are three references to Figure 1 here and the paragraph is really hard to understand; clarify why or if this is intentional.

### Time lags between vertical horse and rider upper-body displacements

- **Line 177:** This is more of a general comment: analyzing individual differences between left- and right-diagonal rising would be interesting.
- **Line 181, 187:** You previously used the term “forelimb.” Ensure consistent terminology throughout.

### Statistics

- **Line 219:** Provide the SPSS version and exact procedure used, including the method for degrees of freedom. SPSS often offers multiple options for this and this might be crucial for the reproducibility of the study.
- **Line 222:** I am not sure if I understand this concept correctly. Please clarify why “horse” was included as a random factor—was each horse measured multiple times?
- **Line 223:** Stride time is listed as both a fixed factor and a covariate. Are you modeling it as both, or only as a covariate? Explain the biological or methodological reasoning behind this.
- **Line 224–225:** Why use p = 0.1 as the cutoff for interaction terms? I know this is the most common approach, but it still should be supported by references or additional rationale. Also consider using AIC/BIC rather than p-value–based stepwise selection.
- **Line 225:** Why is “final” in quotation marks? Are these models not strictly final?
- **Line 226:** After removing non-significant interactions, did you re-evaluate the main effects?
- **Line 226–227:** Was examining histograms the only method used for checking normality? For example, plotting residuals vs. fitted values can additionally help assess homoscedasticity. Were the data normally distributed, or did you use any transformations? Consider including these plots in the supplement.
- Provide the formulas for the final models to enhance clarity.

# Results

## Time offsets between the horses’ stance and flight

- **Lines 230–235 and 236–241:** These sections repeat some of the same information; consider merging them.
- **Line 231 & 237**: Instead of listing the exact p-values, you could indicate they exceeded your threshold (p > 0.1), which ties back to your Methods section discussion.
- **Line 233 & 239:** Provide confidence intervals for the percentage changes.
- Figure 3 is not referenced in the text; make sure to refer to it.

## Horse movement symmetry

- **Line 250:** A single-sentence paragraph appears awkward. After reviewing the entire section, you could merge this into a single concise paragraph, summarizing all significant results without subdividing by body part. Alternatively, consolidate the findings into a table.

### Withers

- **Line 270:** You mention post-hoc pairwise comparisons with Bonferroni corrections, but this was not covered in the Methods. Include it there for clarity (at least in the Supplement).

### Sacrum

- No specific changes noted here but ensure consistency with previous sections.
- **Line 311 (Figure 4):** Presenting withers and sacrum distributions on different scales might improve clarity. Currently, the results for withers and sacrum are less readable due to scale differences between them and the poll.
- **Line 312 (Figure 4 caption):** Define MinDiff, MaxDiff, and UpDiff in the figure caption so it is self-contained.
- **Line 315 (Figure 4 caption):** If positive values indicate right-side asymmetry and negative values indicate left-side asymmetry, consider presenting the boxplots horizontally (with vertical displacement on the x-axis). This could improve readability.
- **Line 321:** Again, a one-sentence paragraph can feel disjointed.
- Consider moving **Figure 5** to the Supplement if it does not show notable results. Also, using many colors and symbols for surface and position when they’re not quite significant factors might create visual “noise.”

### Time lags between vertical horse and jockey upper-body displacements

- **Line 334 and following:** This section feels somewhat cumbersome. You might merge everything into one paragraph or create a concise summary table. Alternatively, a short summary paragraph could be added, though that might further lengthen the manuscript.
- **Line 365 (Figure 6) :** This figure could be made more readable. For instance:
  - Shorten x-axis labels. Since you indicate that “A” is stance and “B” is flight, you needn’t repeat them in the labels. Similarly, “TDiff” is common to all labels and could be moved to the figure caption.
  - Move jockey body part indicators to the first column.
  - Add a horizontal dashed line at y = 0.

### Displacement maximum offsets (horse mid-flight)

- **Line 372 and following:** As with previous paragraphs, consider summarizing only the significant results in a concise format.

# Discussion

General remarks:

- Organizing the discussion around the factors of interest (surface, jockey position) rather than strictly by outcome might be more interesting and engaging.
- Consider discussing the potential impact of horse age. For instance, a yearling or two-year-old might behave differently from an experienced, retired racehorse. Future research could explore this.
- Elaborate on why there was no apparent effect of surface on movement asymmetry, especially in the context of lines 55–72 of the Introduction.
- You need to address the small sample as one of the main limitations of your study.

Specific remarks:

- **Line 418:** Instead of limiting the study’s application to UK training centers, you could generalize since riding surfaces and styles are fairly standardized across many countries (unless you need specific references for a funding requirement).
- **Line 422:** “e.g. between Hyde Park Barracks and Horse Guards, London” is very specific. Remove it unless it serves a funding or sponsor requirement.
- **Line 427:** Reference [27] should appear at the end of the sentence?
- **Line 433–435:** Is a 1.5% difference biologically relevant? Consider discussing whether this is meaningful in practical terms.

## Horse movement asymmetry

- **Line 468–471:** Technical details of the analysis may not be necessary here.
- **Line 486–487, in relation to 498–500**: Good observation regarding the jockey’s hand position compared to dressage riders. Typically, jockeys rest their hands on or near the horse’s neck with reins crossed, so the effect of the hand movement might be limited.
- **Line 501–508:** This is arguably the main takeaway: rising trot does affect movement symmetry. You may wish to emphasize this point more strongly.
- **Line 526–530:** Again, note that stirrup length could significantly influence these findings, as it alters the rider’s center of gravity.

## Horse–jockey co-ordination dynamics

- **Line 558:** Are differences of 2.8–4.5% meaningful? Explain the biological or functional importance. If it is minor, clarify that as well.

# Conclusions

- **Line 615:** You have consistently called the surface “artificial,” so there is no need to rename it “synthetic” here. Maintain consistent terminology.

# References

- Check **reference No. 24**: the journal is labeled as “Science (80-)” which is a common Mendeley quirk. Correct this.

# Supplementary Material

- **Table S1, S5:** Use “p-value” instead of “Sig.” for clarity and consistency.
- **Figure S1:** These results seem more interesting than, for example, Figure 6, yet it is in the Supplement. Regardless, you should discuss it briefly in the main text so readers know its importance.
