# Supplementary material for: Effects of jockey position and surfaces on horse movement asymmetry and horse-jockey synchronisation during trotting exercise
Source: PLoS One. 2025 Jun 2;20(5):e0324753. doi: 10.1371/journal.pone.0324753 (PMC12129221; doi:10.1371/journal.pone.0324753)
Supplement: Attachment — Submitted filename: Response to Reviewers_Final.docx [file pone.0324753.s005.docx]

**Response to Reviewers**

**Reviewers' comments:**

Reviewer's Responses to Questions

**Comments to the Author**

1. Is the manuscript technically sound, and do the data support the conclusions?

The manuscript must describe a technically sound piece of scientific research with data that supports the conclusions. Experiments must have been conducted rigorously, with appropriate controls, replication, and sample sizes. The conclusions must be drawn appropriately based on the data presented.

Reviewer #1: Yes

Reviewer #2: Partly

2. Has the statistical analysis been performed appropriately and rigorously?

Reviewer #1: Yes

Reviewer #2: Yes

3. Have the authors made all data underlying the findings in their manuscript fully available?

The [PLOS Data policy](https://eur01.safelinks.protection.outlook.com/?url=http%3A%2F%2Ftrack.editorialmanager.com%2FCL0%2Fhttp%3A%252F%252Fwww.plosone.org%252Fstatic%252Fpolicies.action%2523sharing%2F1%2F010f019449f1c08e-d0238d1d-3176-49b2-ad08-d8da712705ee-000000%2FEOtPmMdbfiaCCd6zNE_GBEJULr4e0Au07JOZKFCiNEw%3D192&data=05%7C02%7Ckhoran%40rvc.ac.uk%7Ce56c81e44d04464bc9cb08dd307e6c64%7C45a3be7c94024fbead438d1faebfb42d%7C0%7C1%7C638720041743286437%7CUnknown%7CTWFpbGZsb3d8eyJFbXB0eU1hcGkiOnRydWUsIlYiOiIwLjAuMDAwMCIsIlAiOiJXaW4zMiIsIkFOIjoiTWFpbCIsIldUIjoyfQ%3D%3D%7C0%7C%7C%7C&sdata=7x3aWoPuquMOz3rTzJI9jgkH29Me5IRhzcvSGircF1c%3D&reserved=0) requires authors to make all data underlying the findings described in their manuscript fully available without restriction, with rare exception (please refer to the Data Availability Statement in the manuscript PDF file). The data should be provided as part of the manuscript or its supporting information, or deposited to a public repository. For example, in addition to summary statistics, the data points behind means, medians and variance measures should be available. If there are restrictions on publicly sharing data—e.g. participant privacy or use of data from a third party—those must be specified.

Reviewer #1: Yes

Reviewer #2: No

4. Is the manuscript presented in an intelligible fashion and written in standard English?

PLOS ONE does not copyedit accepted manuscripts, so the language in submitted articles must be clear, correct, and unambiguous. Any typographical or grammatical errors should be corrected at revision, so please note any specific errors here.

Reviewer #1: Yes

Reviewer #2: Yes

5. Review Comments to the Author

Please use the space provided to explain your answers to the questions above. You may also include additional comments for the author, including concerns about dual publication, research ethics, or publication ethics. (Please upload your review as an attachment if it exceeds 20,000 characters)

**Reviewer #1:** A really interesting pilot study manuscript that I thought was well written except for a few minor typos throughout. Examples below.

*We would like to thank the reviewer for their positive feedback and have made all corrections suggested.*

In your abstract, you report "jockeys reduced poll asymmetry", but you only used one jockey.

*This has been amended.*

Last sentence of your introduction: "when the jockey imposes and uneven biphasic load" - did you mean "an"?

*Yes, this has been corrected.*

Third sentence in "Horse Movement asymmetry": you have an unnecessary comma between "previous study".

*This has been amended.*

**Reviewer #2:** Dear Authors,

Thank you for submitting your very interesting study. I have taken the liberty of numbering the lines in your manuscript and will refer to them in my comments. I enjoyed reading your paper; however, I found it somewhat lengthy in places, which made it occasionally challenging to follow. I am also somewhat concerned about your sample size, though I acknowledge that including multiple strides may help compensate for this limitation. Please find my comments attached.

*We would like to thank the reviewer for their detailed and helpful review of our manuscript. Please find below our point-by-point response to each of their suggestions.*

Title

The current title seems a bit extensive given that your findings indicate surfaces have minimal impact on asymmetry. I recommend rephrasing it to “Effects of the surfaces and jockey position on horse movement asymmetry and horse–jockey synchronization during trotting exercise”.

*We have amended the title to: “Effects of jockey position and surfaces on horse movement asymmetry and horse-jockey synchronisation during trotting exercise”.*

Abstract

- **Line 15:** Your opening sentence effectively explains the rationale for the study, but it may give the impression that both horses and jockeys are the primary focus. Consider removing “and jockeys,” and perhaps acknowledge the fact that most injuries occur during canter/gallop exercises. For example “Racehorses can incur injuries not only during gallops, but also during routine trotting exercise to access gallop tracks or warm up.”

*We still wish to refer to jockey injuries in our opening sentence because injuries to jockeys, as well as their horses, during access to gallops was a motivation for this investigation. However, we have adjusted the sentence slightly as per the reviewer’s suggestion and it is now as follows:*

*“Racehorses and jockeys can incur injuries, not only during gallops, but also during routine trotting exercise to access gallop tracks or warm-up.”*

- **Line 19:** Here you use the word “turf”, but you call this surface “grass” throughout the whole manuscript. Replace “turf” so it’s not confusing.

*We have made this amendment to improve consistency.*

- **Line 22–25:** This sentence feels slightly out of place in the abstract. Consider introducing the technical parameters of inertial sensors (in fewer details if you prefer) earlier (around line 17) to improve flow.

*We have amended and condensed the opening section of the abstract to the following:*

*“… This study used inertial sensing technology (XSens MTw sensors) and linear mixed models to quantify and determine the significance (p≤0.05) of jockey position (‘rising’ versus ‘two-point seat’) and surface type (artificial, grass and tarmac) on: 1) time offsets between stance and flight phases; 2) horses’ vertical upper body movement asymmetry and 3) time lags (in % of stride time) between horse and jockey maximum and minimum vertical displacements. Six ex-racehorses were recruited on a convenience basis from the British Racing School and were ridden by one jockey.”*

- **Line 28–29:** The abbreviations “MinDiff,” “MaxDiff,” and “UpDiff” should be defined if used in the abstract. However, you might consider removing them (and the associated p-values) from this sentence entirely to keep the abstract concise.

*We have decided to remove these terms but feel it relevant to keep the p values in the abstract.*

- **Line 40:** Adding a single sentence summarizing the results would enhance the abstract’s readability.

*We have added the following sentence: “In summary, jockey position had a greater impact on horse movement asymmetry and horse-jockey synchronisation than surface type.”*

Introduction

- **Line 42:** Specify whose safety you are referring to—jockeys, racehorses, or both.

*We have added the word “both”.*

- **Line 51 and 53:** Using seven or six references to support one statement may be excessive. A common guideline is a maximum of three references per statement. Consider reducing the number of references in this section.

*We have considered the references cited and feel they are appropriate to include here.*

- **Lines 55–72:** This paragraph is somewhat lengthy. Consider shortening by removing some details that are not essential to your analysis.

*In light of this comment, we have restructured and reduced the length of this paragraph. This section now reads as follows:*

*“… Nevertheless, it is recognized that rider kinematics can adjust to accommodate changes in translational and rotational upper body movements of their horse [15], and vice versa [16,17]. Therefore, if hoof-ground interactions influence the upper body movement of the horse through alterations to the magnitude, direction and origin of ground reaction force, they are expected to have relevance for horse-rider stability and movement efficiency. For example, excessive hoof slip may lead to biomechanical instabilities that prevent a horse from being able to support its centre of mass, thus causing it to stumble or fall, and potentially sustain an injury to its musculoskeletal system [18]. Even small adjustments at the shoe-surface interface have the potential to influence a horse’s gait; for example, a unilateral hindlimb road nail, protruding 3 mm from a horseshoe surface was shown to induce small pelvic movement asymmetry in horses trotting on tarmac [19]. This effect was thought to reflect a reduction in hoof slip caused by the nail, and therefore increased time for weight-bearing and generation of propulsive force during stance. Jockey injuries are very often linked to horse falls [21], therefore factors that influence a horse’s gait may have repercussions for the risk of jockey injury and falls. This may be particularly catastrophic if horse slipping incidents coincide with passing vehicular traffic.”*

Materials and Methods

Horse and rider participants

- **Line 96:** Include the horses’ ages (e.g., a retired 4-year-old may behave differently than a 15-year-old). Also provide how long they raced and how long they have been retired.

*We have added the following details on the horses to this paragraph: “A convenience sample of six retired Thoroughbred racehorses in regular work and utilised for jockey education at the British Racing School (BRS) in Newmarket, UK, was included in this study. These horses are no longer involved in competitive racing but are fit and work under conditions similar to those encountered during active race training. The horses had ages between 7 and 19 years, their masses ranged from 510 to 580 kg, and their heights ranged from 16 to 17 hh (1.63 to 1.73 m).”*

- **Line 96–97:** Since the subject of this sentence is “sample,” the verb should be singular—“was included in this study,” rather than “were included.”

*This has been corrected.*

- **Line 97–98:** Consider mentioning the jockey’s experience. How long have they ridden racehorses? Were they a BRS student? Have they ridden in races, or only as a work rider? This information is especially relevant to your discussion in lines 548–550.

We *have added the following details on the jockey to this paragraph: “The same jockey rode all horses and had a body mass of approximately 60 kg. The jockey had been chosen to ride the horses on 22/05/2021 by the BRS management team. They have a category A and point-to-point license and approximately ten years of experience in the racing industry. They are riding racehorses daily and as such were fit to participate in multiple trials with all horses and they did not become fatigued during the data collection.”*

- If possible, include details on the saddle type (I imagine it was a racing exercise saddle), including weight. The saddle can significantly influence rising trot technique. Similarly, provide information on stirrup length, since this can affect the rider’s center of gravity.

*We have noted that they rode in a racing exercise saddle. We do not have details on the saddle mass so do not include this. Stirrup length was not quantified so cannot be provided.*

Equipment

- This section is the only one that’s numbered. Make sure you maintain consistent formatting.

*We have added line numbers to the whole document now.*

- Line 101: Maybe add the word “each” to underline that 16 g is the mass of a single sensor, not the collection of all six.

*We have made this suggestion.*

- Line 108: For reproducibility, consider adding basic information about the computer used (operating system, CPU, RAM).

*We have added that it was a Windows 10 Pro computer.*

Trial conditions

- Although optional, including illustrative photos or diagrams of rising trot vs. two-point seat, as well as left- vs. right-diagonal rising trot (similar to your current Figure 1), could help non-specialist readers.

*We have decided not to introduce another figure at this stage as we feel our definition of rising and two-point seat is sufficient, given these are very common equestrian terms.*

- **Line 120-122:** I understood your point only after reading the whole manuscript, so you may want to rephrase these sentences. Because of the fact the you describe the softness of the artificial and grass (line 120), the following sentence (lines 121-122) sounds a bit like the tarmac was used only as merely a “reference surface” for in-hand trotting.

*Thank you for drawing our attention to this. On line 117 it had been specified that “Trials were performed on tarmac, an artificial training track (Martin Collins Activ Track) and a grass training track.”*

*In addition, we have now added the following to further improve clarity “In addition to the ridden trials on the artificial surface, grass and tarmac, to acquire reference movement symmetry values, horses were additionally trotted in-hand in a straight line over the tarmac…”*

Data processing and statistics

Overview

- **Line 130–131:** Did you consider any interpolation methods other than linear, such as cubic splines or Fourier-based fitting? These might be more appropriate for this kind of wavy function.

*There will be little effect of interpolation method for “oversampled” signals such as the ones here. In trot, the fundamental frequency of upper body movement signals is either stride frequency (e.g. for mediolateral movement) or twice stride frequency (e.g. for vertical movement). The original signal was sampled at 60 Hz. Therefore, as the horses had a stride frequency for trot in the region of 1.5 Hz, one would need >3 Hz sample rate. Given we used a sample rate of 60 Hz there is little to be gained from more sophisticated interpolation methods and this supported by the following publication on sample rate:* “*Pfau, T. and Reilly, P., 2021. How low can we go? Influence of sample rate on equine pelvic displacement calculated from inertial sensor data. Equine veterinary journal, 53(5), pp.1075-1081.”*

Time between stance and flight phases

- There are three references to Figure 1 here and the paragraph is really hard to understand; clarify why or if this is intentional.

*We have restructured this paragraph, and we now just include one reference to Figure 1.*

Time lags between vertical horse and rider upper-body displacements

- **Line 177:** This is more of a general comment: analyzing individual differences between left- and right-diagonal rising would be interesting.

*Yes, this is interesting and particularly in the context of underlying horse asymmetry. However, it would be difficult to assess in this small sample set.*

- **Line 181, 187:** You previously used the term “forelimb.” Ensure consistent terminology throughout.

*We have corrected front limb to forelimb in both cases.*

Statistics

- **Line 219:** Provide the SPSS version and exact procedure used, including the method for degrees of freedom. SPSS often offers multiple options for this and this might be crucial for the reproducibility of the study.

*We have added that it was “IBM SPSS statistical software (version 28)”.*

*We have also added the following sentence “In all cases, models adopted the Restricted Maximum Likelihood method and the Satterthwaite approximation for the degrees of freedom.”*

- **Line 222:** I am not sure if I understand this concept correctly. Please clarify why “horse” was included as a random factor—was each horse measured multiple times?

*Yes, each horse completed multiple trials, as indicated under the ‘Trial Conditions’ section.*

*We have added that we “were not interested in the effects of individual horses” to this sentence.*

- **Line 223:** Stride time is listed as both a fixed factor and a covariate. Are you modeling it as both, or only as a covariate? Explain the biological or methodological reasoning behind this.

*We have added the following sentence: “Stride time was included as a covariate (as well as a fixed factor) because we were also interested in its numerical relationship with the outcome parameters; for example, stride time is typically related to speed [34] and it seemed important to establish whether the investigated variables would increase or decrease with speed (stride time).”*

- **Line 224–225:** Why use p = 0.1 as the cutoff for interaction terms? I know this is the most common approach, but it still should be supported by references or additional rationale. Also consider using AIC/BIC rather than p-value–based stepwise selection.

*We have added that “We chose this method in agreement with previous studies [e.g. 5,18].”*

- **Line 225:** Why is “final” in quotation marks? Are these models not strictly final?

*The quotation marks have been removed.*

- **Line 226:** After removing non-significant interactions, did you re-evaluate the main effects?

*Yes, we have now clarified this by adding the following sentence: “This meant that the single fixed factors were reevaluated alongside only the interaction terms that had been significant in the preliminary models.”*

- **Line 226–227:** Was examining histograms the only method used for checking normality? For example, plotting residuals vs. fitted values can additionally help assess homoscedasticity. Were the data normally distributed, or did you use any transformations? Consider including these plots in the supplement.

*It is common practice to visually inspect the distribution of residuals, so we followed this approach and noted that the residuals were normally distributed. We have now added that “no data transformations were deemed necessary”.*

- Provide the formulas for the final models to enhance clarity.

*Apologies, but we are not quite sure what the reviewer is after here so there are no additional formulas included.*

Results

Time offsets between the horses’ stance and flight

- **Lines 230–235 and 236–241:** These sections repeat some of the same information; consider merging them.

*We have merged this information.*

**Line 231 & 237**: Instead of listing the exact p-values, you could indicate they exceeded your threshold (p > 0.1), which ties back to your Methods section discussion.

*We have combined the information on the preliminary models for the stance and flight phases.*

- **Line 233 & 239:** Provide confidence intervals for the percentage changes.

*We now provide 95% confidence intervals for the percentage changes.*

- Figure 3 is not referenced in the text; make sure to refer to it.

*We have now referred to Figure 3 in the main text (opening sentence of the results).*

Horse movement symmetry

- **Line 250:** A single-sentence paragraph appears awkward. After reviewing the entire section, you could merge this into a single concise paragraph, summarizing all significant results without subdividing by body part. Alternatively, consolidate the findings into a table.

*Unlike the section on “Time offsets between the horses’ stance and flight” these paragraphs on movement asymmetry results are harder to combine. This is because the final models included different factors and we wish to keep this detail in the manuscript. For clarity however, the significant factors are now additionally summarised in Figure 4.*

Withers

- **Line 270:** You mention post-hoc pairwise comparisons with Bonferroni corrections, but this was not covered in the Methods. Include it there for clarity (at least in the Supplement).

*In the ‘Statistics’ section we now state:* *“All post-hoc pairwise comparisons included Bonferroni correction”.*

Sacrum

- No specific changes noted here but ensure consistency with previous sections.
- **Line 311 (Figure 4):** Presenting withers and sacrum distributions on different scales might improve clarity. Currently, the results for withers and sacrum are less readable due to scale differences between them and the poll.

*As per the reviewers suggestion, we have adjusted the y axis scales so they are specific to each panel within the plot.*

- **Line 312 (Figure 4 caption):** Define MinDiff, MaxDiff, and UpDiff in the figure caption so it is self-contained.

*We have added these definitions to the caption.*

- **Line 315 (Figure 4 caption):** If positive values indicate right-side asymmetry and negative values indicate left-side asymmetry, consider presenting the boxplots horizontally (with vertical displacement on the x-axis). This could improve readability.

*We have added arrows to the plot indicating the direction of right and left sided asymmetry.*

*To improve clarity further, we have now added symbols on Figure 4 to indicate which main effects influenced the data.*

- **Line 321:** Again, a one-sentence paragraph can feel disjointed.

*We have moved this sentence to the end of this section.*

- Consider moving **Figure 5** to the Supplement if it does not show notable results. Also, using many colors and symbols for surface and position when they’re not quite significant factors might create visual “noise.”

*We prefer to leave the colour scheme as it is because it allows the reader to visually assess the data and potential influencing factors.*

Time lags between vertical horse and jockey upper-body displacements

- **Line 334 and following:** This section feels somewhat cumbersome. You might merge everything into one paragraph or create a concise summary table. Alternatively, a short summary paragraph could be added, though that might further lengthen the manuscript.

*To improve clarity, we have now added symbols on Figure 6 to indicate which main effects influenced the data.*

- **Line 365 (Figure 6) :** This figure could be made more readable. For instance:
  - Shorten x-axis labels. Since you indicate that “A” is stance and “B” is flight, you needn’t repeat them in the labels. Similarly, “TDiff” is common to all labels and could be moved to the figure caption.

*We have now condensed the x-axis labels.*

- Move jockey body part indicators to the first column.

*We are not quite sure what the reviewer would like us to change here. As there is a part A and part B to this figure we label the relevant anatomical parts for the jockey on each sub-figure.*

- Add a horizontal dashed line at y = 0.

*This has been added.*

Displacement maximum offsets (horse mid-flight)

- **Line 372 and following:** As with previous paragraphs, consider summarizing only the significant results in a concise format.

*To improve clarity, we have now added symbols on Figure 6 to indicate which main effects influenced the data.*

Discussion

General remarks:

- Organizing the discussion around the factors of interest (surface, jockey position) rather than strictly by outcome might be more interesting and engaging.

*We have completely restructured the discussion, so there are now two main sections around the themes of ‘surface’ and ‘jockey position’, as per the reviewer’s suggestion.*

- Consider discussing the potential impact of horse age. For instance, a yearling or two-year-old might behave differently from an experienced, retired racehorse. Future research could explore this.

*We have added the following sentence to our discussion:*

*“It would also be interesting to evaluate the role of horse age on the data as a yearling or two-year-old might behave differently from an experienced, retired racehorse.”*

- Elaborate on why there was no apparent effect of surface on movement asymmetry, especially in the context of lines 55–72 of the Introduction.

*We have added the following to the new ‘Surface’ section of our discussion:*

“*In addition, although there may be differences in upper body range of motion at trot on different surface types (Horan et al., in prep.), the fact that there was a consistent bilateral change in the hoof-ground interaction in the two halves of the trot cycles may also explain why there was little change in asymmetry.”*

- You need to address the small sample as one of the main limitations of your study.

*We have added the following statement:*

*“It would also be beneficial to observe the gait kinematics in a larger sample size as the low sample number here is a limitation of the current study.”*

Specific remarks:

- **Line 418:** Instead of limiting the study’s application to UK training centers, you could generalize since riding surfaces and styles are fairly standardized across many countries (unless you need specific references for a funding requirement).

*We have deleted “…including Newmarket, Lambourn and Northern England (such as Middleham)”*

- **Line 422:** “e.g. between Hyde Park Barracks and Horse Guards, London” is very specific. Remove it unless it serves a funding or sponsor requirement.

*We have removed this.*

- **Line 427:** Reference [27] should appear at the end of the sentence?

*We have deleted this reference.*

- **Line 433–435:** Is a 1.5% difference biologically relevant? Consider discussing whether this is meaningful in practical terms.

*We have added a comment at the end of this paragraph:*

*“However, the biological relevance of this difference requires further investigation.”*

Horse movement asymmetry

- **Line 468–471:** Technical details of the analysis may not be necessary here.

*We have deleted “with Bonferroni correction” as this is now included in the methods.*

- **Line 486–487, in relation to 498–500**: Good observation regarding the jockey’s hand position compared to dressage riders. Typically, jockeys rest their hands on or near the horse’s neck with reins crossed, so the effect of the hand movement might be limited.

*We have not altered anything here.*

- **Line 501–508:** This is arguably the main takeaway: rising trot does affect movement symmetry. You may wish to emphasize this point more strongly.

*We now open our new “Surface” section with “Overall, the effect of surface on the time offsets between stance and flight phases, horses’ vertical upper body movement asymmetry, and the time lags between horse and jockey maximum and minimum vertical displacements was limited compared to jockey position effects.”*

*Our abstract also now includes the following sentence: “**In summary, jockey position had a greater impact on horse movement asymmetry and horse-jockey synchronisation than surface type.”*

- **Line 526–530:** Again, note that stirrup length could significantly influence these findings, as it alters the rider’s center of gravity.

*We have added the following sentence:*

*“In addition, the shorter stirrup lengths used by jockeys compared to dressage riders may be accountable for some of the differences in findings between the present study and previous work, because changes to stirrup length will alter the position of a rider’s centre of mass.”*

Horse–jockey co-ordination dynamics

- **Line 558:** Are differences of 2.8–4.5% meaningful? Explain the biological or functional importance. If it is minor, clarify that as well.

*We have added the following sentence at the end of this section:*

*“In addition, at present it is not possible to evaluate the functional significance of the observed time-offsets between horse and jockey displacement minima and maxima and their bearing on horse-jockey safety.”*

Conclusions

- **Line 615:** You have consistently called the surface “artificial,” so there is no need to rename it “synthetic” here. Maintain consistent terminology.

*We have corrected this.*

References

- Check **reference No. 24**: the journal is labeled as “Science (80-)” which is a common Mendeley quirk. Correct this.

*This has been corrected.*

Supplementary Material

- **Table S1, S5:** Use “p-value” instead of “Sig.” for clarity and consistency.

*We have replaced “Sig.” With “P value” in tables S1, S5 and S9.*

- **Figure S1:** These results seem more interesting than, for example, Figure 6, yet it is in the Supplement. Regardless, you should discuss it briefly in the main text so readers know its importance.

*We have added the following to the methods on Figure S1:*

*“The distribution of stride times across trial conditions in provided in Fig. S1.”*

*We have also added the following comment on Figure S2:*

*“In addition, we observed that the angle of the jockey’s back seemed to adopt a more consistent orientation in rising trot compared to two-point seat (Figure S2).”*
